# Cross Analysis of Genomic-Pathologic Features on Multiple Primary Hepatocellular Carcinoma

**DOI:** 10.3389/fgene.2022.846517

**Published:** 2022-06-20

**Authors:** Fei Ren, Depin Wang, Xueyuan Zhang, Na Zhao, Xiaowen Wang, Yu Zhang, Li Li

**Affiliations:** ^1^ High Performance Computer Research Center, Institute of Computing Technology, Chinese Academy of Sciences, Beijing, China; ^2^ Zhijian Life Co. Ltd., Beijing, China; ^3^ Department of Medical Oncology and Radiation Sickness, Peking University Third Hospital, Beijing, China; ^4^ Department of Oncology, Peking University International Hospital, Beijing, China

**Keywords:** hepatocellular carcinoma, whole exome sequencing, whole slide images, molecular profiling and subtyping, hepatitis B virus

## Abstract

Hepatocellular carcinoma (HCC) is a prevalent malignancy cancer worldwide with a poor prognosis. Hepatic resection is indicated as a potentially curative option for HCC patients in the early stage. However, due to multiple nodules, it leads to clinical challenges for surgical management. Approximately 41%–75% of HCC cases are multifocal at initial diagnosis, which may arise from multicentric occurrence (MO-HCC) or intrahepatic metastasis (IM-HCC) pattern with significantly different clinical outcomes. Effectively differentiating the two mechanisms is crucial to prioritize the allocation of surgery for multifocal HCC. In this study, we collected a multifocal hepatocellular carcinoma cohort of 17 patients with a total of 34 samples. We performed whole-exome sequencing and staining of pathological HE sections for each lesion. Reconstruction of the clonal evolutionary pattern using genome mutations showed that the intrahepatic metastogenesis pattern had a poorer survival performance than independent origins, with variants in the TP53, ARID1A, and higher CNV variants occurring more significantly in the metastatic pattern. Cross-modality analysis with pathology showed that molecular classification results were consistent with pathology results in 70.6% of patients, and we found that pathology results could further complement the classification for undefined patterns of occurrence. Based on these results, we propose a model to differentiate the pattern of multifocal hepatocellular carcinoma based on the pathological results and genome mutations information, which can provide guidelines for diagnosing and treating multifocal hepatocellular carcinoma.

## Introduction

Liver cancer is ranked as the sixth most common malignancy cancer, and its incidence is rising (Sung et al., 2021). Hepatocellular carcinoma (HCC) is the most common form of liver cancer, accounting for approximately 90% of liver cancer cases (Llovet et al., 2021). Roughly 41%–75% of patients with HCC present with multiple intrahepatic tumors (Miao et al., 2014; Vogel et al., 2018). Despite there existing standardized guidelines for multifocal HCC and indications for surgical resection, surgical suggestions for individual patients remain complicated owing to the difficulty of accurately predicting future tumor progression. These uncertainties for the recurrence of primary lesions or metastatic possibility provide challenges to the prognosis after surgery for individual patients (Viganò et al., 2019).

Multifocal HCC may arise synchronously or metachronously as a separate primary tumor (multicentric occurrence) or develop due to intrahepatic metastases from the same primary cancer (Baffy, 2015). Since the prognosis of hepatocellular carcinoma patients under these two types varies greatly, it is crucial to construct the correct diagnostic approach for these patients. Several assessment methods, including pathological examination, integration of hepatitis B virus (HBV) DNA by PCR and DNA blot analysis, and heterozygosity analysis of DNA microsatellite loci, have been recently developed to distinguish between these two types of multifocal HCC (Study, 2012). However, the combination of molecular and pathological profiling with analytical methods systematically used to distinguish between these two patterns are still lacking.

The advantage and rapid progress of next-generation sequencing, such as whole-exome sequencing (WES), has made it possible to comprehensively characterize the disease mechanisms and altered genes in multiple cancers (Ally et al., 2017; Yan et al., 2018; Nanki et al., 2020). This approach allows the identification of novel molecular markers and the definition of underlying biological mechanisms, thus facilitating the stratification and characterization of cancers (Cortés-Ciriano et al., 2022). In this study, we selected representative patients of HBV-associated multifocal HCC who underwent tumor resection and exhibited a variable postoperative course.

These HCC samples were conducted whole-exome sequencing (WES) to obtain a complete genetic alteration profiling for each patient. We then performed a systematic analysis of integrated genomics and further correlated these with clinic pathological data. We sought to comprehensively unravel the molecular differences between the two multifocal HCC models as well as differences in pathological features and identify molecular markers for diagnostic, prognostic, and potential therapeutic targets to guide the clinical diagnosis and treatment of multifocal hepatocellular carcinoma.

## Methods

### Mutation Analysis

First, we aligned the exome sequencing clean reads against the human reference genome hg19 download from UCSC (http://www.genome.ucsc.edu/) using BWA (Li and Durbin, 2009) with the default parameters. To reduce systematic (non-random) technical error, we applied base quality recalibration with the Genome Analysis Toolkit (GATK) 4.0 (McKenna et al., 2010). The duplicated reads was removed from the alignment files using the Picard tools. Somatic variants, including single nucleotide variants (SNVs) and small insertions and deletions changes (Indels), were detected by Mutect2 of GATK 4.0 on the paired tumor and normal samples. High confidence variants were screened using the criteria of TLOD >10, and then they were annotated by the vcf2maf tool (https://github.com/mskcc/vcf2maf) to obtain nine types of mutations, including “Missense Mutation”, “Nonsense Mutation”, “Nonstop Mutation”, “Splice Site”, “Splice Region”, “In Frame Ins”, “In Frame Del”, “Frame Shift Ins” and “Frame Shift Del” mutations.

### Copy Number Analysis

The bioinformatics tool facet-suite (R package) (Shen and Seshan, 2016) was utilized to detect CNVs on paired sequencing reads of tumor and normal samples from the same patient. We first assessed the copy number of different segments and then filtered those segments with a total copy number greater than twice the DNA ploidy level as the amplification (AMP), and segments with a total copy number equal to zero as deletions (DEL). These AMP or DEL segments were the annotated with genes located in the genome context to obtain gene-level copy number alteration. To summarize total copy number variation at the level of the whole exome, we calculated a CNV score, which is similar to the TMB, simply by multiplying the length of CNV segments by their relative average altered weight.

### Tumor Mutational Burden Analysis

As the predictive biomarker in solid tumors (Wu et al., 2019), the tumor mutational burden (TMB), was calculated for all tumor samples by counting the non-synonymous mutation rate per megabases. We screened nine types of non-silent mutations from the analysis of the vcf2maf annotation tool. Those nine types of variants include “Splice Site”, “Splice Region”, “Missense Mutation”, “Nonstop Mutation”, “Nonsense Mutation”, “Frame Shift Ins” and “Frame Shift Del”, “In Frame Ins”, “In Frame Del”. Then these variants were all counted for TMB calculation, and the values were normalized by the total length of the CDS regions (36 Megabases) covered by the Agient V6 whole exome (Wang et al., 2020).

### Microsatellite Instability and Mutational Signature Analysis

We evaluated the MSI status of the tumor samples with the bioinformatics tool Msisensor (Niu et al., 2014), and screened MSI-H samples with the criteria of an MSIsensor score greater than 20 (Shimozaki et al., 2021). We determined the frequency of 96 mutated triplets per tumor sample based on the distribution of the six substitution patterns (C > A, C > G, C > T, T > A, T > C, T > G) and the neighbor 5′ base and 3′ base (Alexandrov et al., 2013). Together with their frequency, these triplets were summarized to construct a 96 × N mutation type frequency matrix, where N is the number of variants. We took the matrix as the input to determine the 1–30 mutational signatures (Alexandrov et al., 2020) from the Cosmic database (Tate et al., 2019) and to assess the proportion of specific mutational signatures in the samples using the bioinformatics tool DeConstructSig (Rosenthal et al., 2016). 30 mutational signatures were then reduced and classified to mutational signature 1, mutational signature 3, mutational signature 6, mutational signature 10 and others according to their different frequencies in the HCC samples (others represent the less frequently mutated mutational signatures in the HCC samples).

### Phylogenetic Analysis

Phylogenetic analyses were performed to elucidate genes essential for promoting tumor recurrence. We compared mutant variants in samples from different cancer samples, counted unique and shared mutations, and used the common and unique mutations in two cancer samples to construct a phylogenetic tree. The phylograms were inferred using the R Bioconductor package phangorn (Schliep, 2011). Through phylogenetic tree analysis, we were able to identify early driver mutations and *de novo* mutations at different stages, thus providing a comprehensive interpretation of the relationships between different tumors.

### Pathology Image Analysis

Feature extraction of cell nucleus from pathology images mainly includes cancer region labeling, patch segmentation, color normalization, nucleus segmentation, nucleus-level and image-level feature extraction (Cheng et al., 2020): 1) The whole slide images (WSIs) were labeled the cancer region manually. 2) Non-overlapping image tiles with a size of 2048*2048 pixels with a resolution of 0.5 μm per pixel were extracted from Whole Slide Images (WSIs). To remove the bias of different staining procedures, all tiles were normalized based on one reference image using the Macenko normalization method. 3) Use a hierarchical multilevel thresholding approach to segment the nucleus for each tile. 4) Calculate 10 features of each nucleus in each image patch. 5) For the nuclei of all patches in one WSI, each type of nucleus-level features was dissected into 15 image-level features by combining a 10-bin histogram and 5 distribution statistics (mean, std, skewness, kurtosis, and entropy). In total, we calculated 100 image-level features for each whole-slide image.

### Statistical Analysis

We use the student *t* test to compare the difference between two continuous variables. Kaplan-Meier survival analysis was used to obtain survival curves reflecting the differences in prognosis among tumor subtypes. Log-rank test was couducted to assess the correlation. Mann-Whitney *U* test was utilized to analyze the relationship between the two classification variables.

## Results

### Collection of Multifocal HCC Samples

The clinical outcome of patients with HCC undergoing radical surgery are closely related to the number of intrahepatic tumors. The main purpose of this study was to explore genomic and pathological characteristics among the different intrahepatic tumors and discover multiple modality indicators, thus we specified the intrahepatic tumor numbers to be 2. Multifocal HCC samples are collected from Peking University International Hospital and Peking University Third Hospital, and the inclusion criteria is as follows. The tumor satisfies the criteria for surgical indications defined by the Chinese CSCO Guidelines for primary HCC. Postoperative pathology confirmed that the tumor was hepatocellular carcinoma. Tumors were radically resected (R0), and the number of tumors was two. A total of 17 cases of patients met the criteria, and 34 tumor samples were performed whole-exome sequencing.

### Molecular Profiling of Liver Cancer

A total of 17 patients with multifocal liver cancer were recruited for our study, with two cancer foci collected per patient. Sixteen of them were male, one was female, and the cohort’s median age was 45 years (distribution 43–67 years). The median follow-up time was 42 months. WES was performed on 34 tumor samples and paired samples of FFPE specimens, with a average 200× coverage depth for both the tumor and normal samples. The detailed clinical and pathological information of all patients used in this research is given in Supplementary Table S1.

**FIGURE 1:**
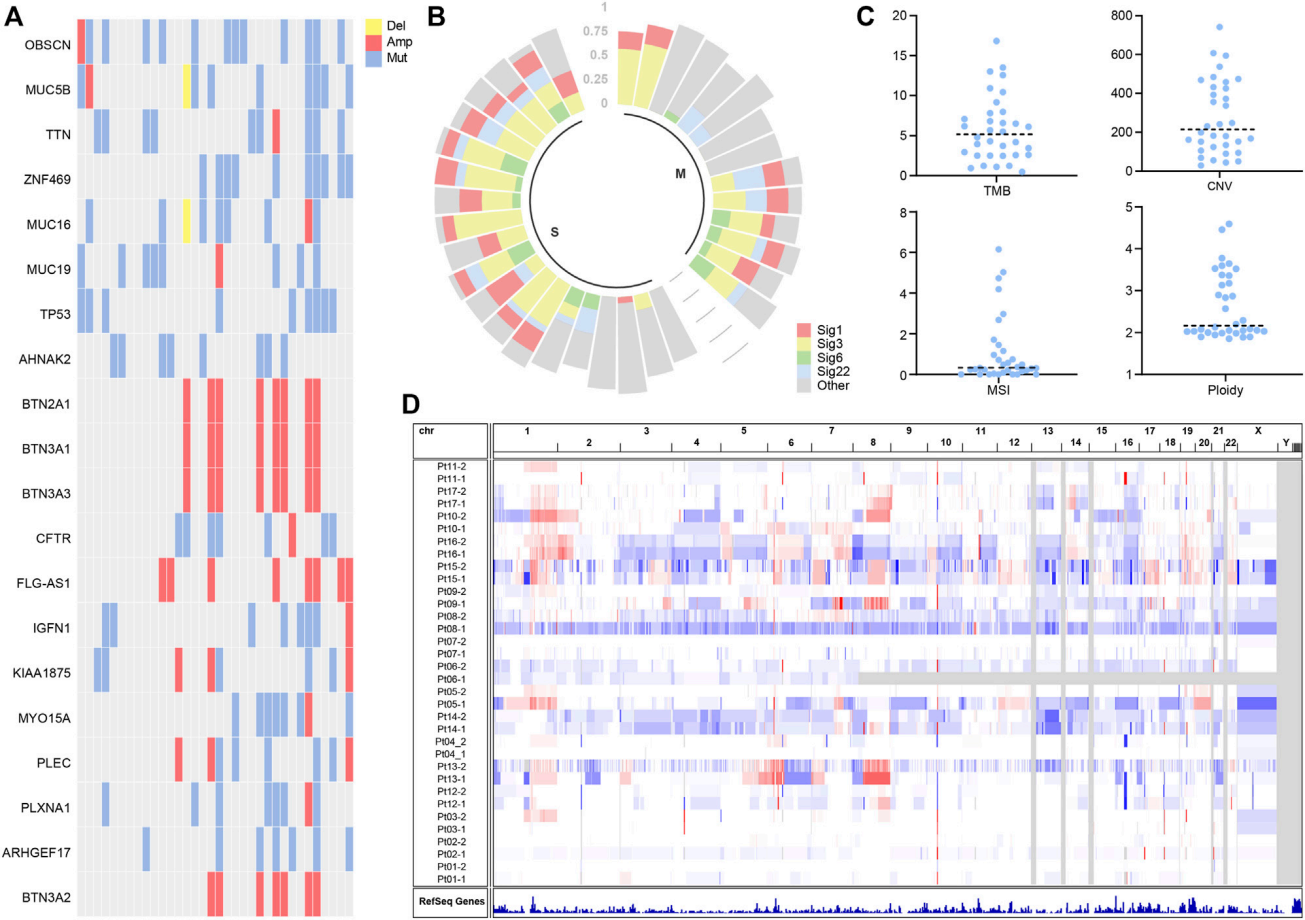
**(A)** Landscapes of frequently mutated genes in liver cancer. **(B)** Mutation signature of liver cancer. **(C)** Characterization statistics of TMB, CNV, MSI, and Ploidy. **(D)** Copy number alterations in liver cancer.

To disentangle somatic mutations and molecular characteristics of multifocal hepatocellular carcinoma, mutation analysis of 34 tumor samples identified 7,752 individual mutations, including 6,378 single nucleotide variants (SNVs) and 1,374 small insertions and deletions changes (Indels) (Supplementary Table S2). The mean number of non-synonymous mutations per sample was 77 (range: 10–176), corresponding to 3.5 non-synonymous mutations per Megabyte (Mb), comparable to the TMB in the TCGA cohort. To explore potential driver mutations in patients, we summarized multiple genes with the highest mutation frequency (Figure 1A). The most commonly mutated genes in these patients were OBSCN, MUC5B, TTN, ZNF469, MUC16, TP53, with VAF greater than 25%. The frequency of TP53 variants is comparable to that observed in the TCGA cohort. Deletions were not widespread in genes with high mutation rates, while BTN2A1, BTN3A1, BTN3A3, BTN3A2, and FLG-AS1 were amplified in several samples.

Mutation signature analysis showed mutation signature 1, 3, 6, and 22 to be more prevalent in patients (Figure 1B). According to published reports (Koh et al., 2021), Signature 6 is associated with DNA mismatch repair defects and MSI tumors. Signature 1 is associated with age at cancer diagnosis and has been detected in most types of cancer samples. Signature 3 is associated with homologous repair and correlates with BRCA gene function. Signature 22 has been found in urothelial (renal pelvis) carcinoma and liver cancers.

We characterize molecular features of TMB, CNV, MSI, and Ploidy. It is shown that the median TMB was around 5.15 (Figure 1C), and the median CNV was assessed at 214.9 (Figure 1C). The MSI analysis showed that most liver cancer samples had low MSISensor scores, all less than 10 (Figure 1C). Most of the samples had a ploidy around 2 (Figure 1C). The copy number of chromosomes has more amplification events on chromosomes 1 and 8 (Figure 1D).

### Identification of Hepatocarcinogenesis Pattern by Genomic Signature

**FIGURE 2:**
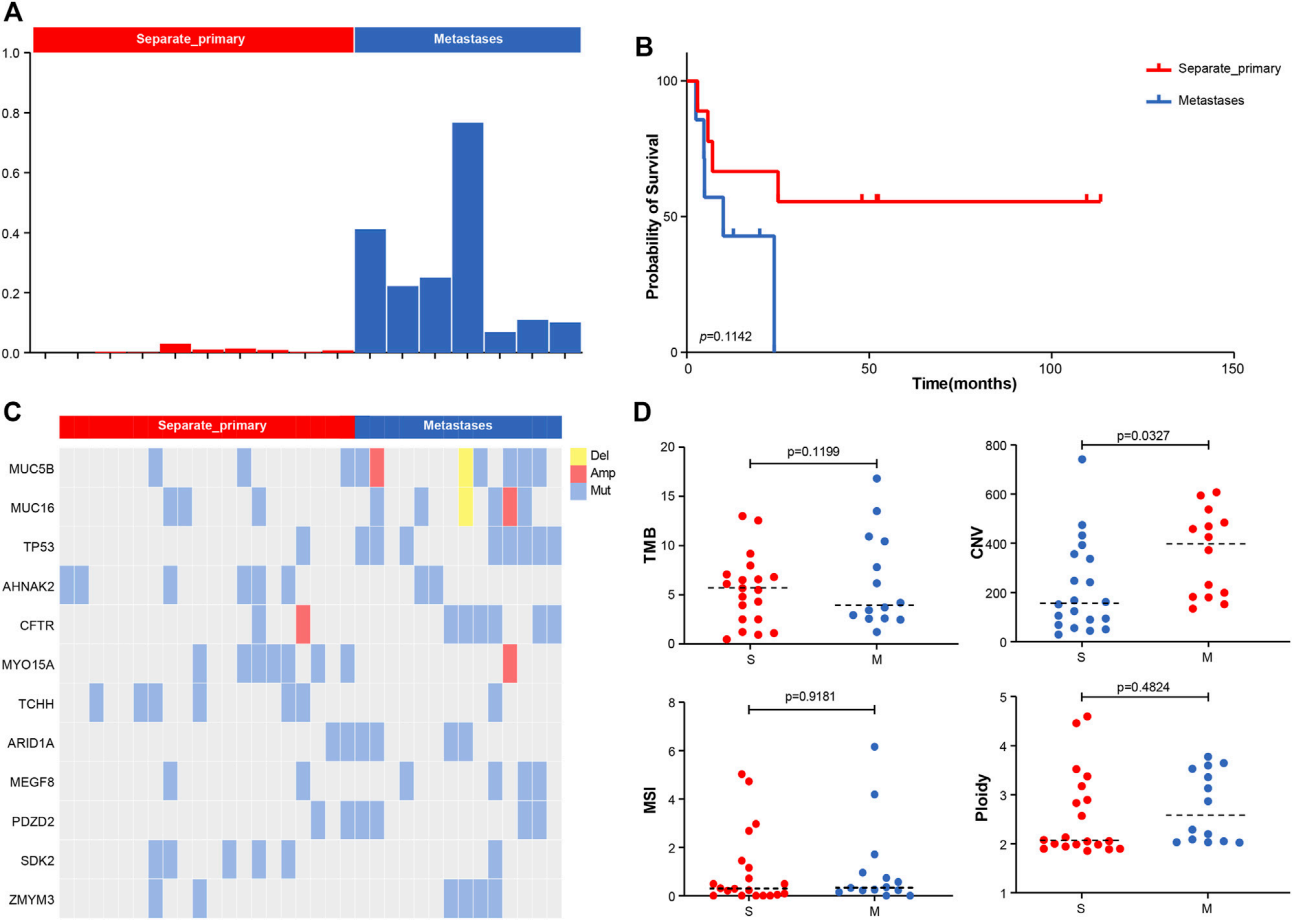
**(A)** Molecular typing strategy of two cancer subtypes. **(B)** Associations between cancer types and OS. **(C)** Comparison of the prevalence of altered genes between two cancer subtypes. **(D)** Comparison of TMB, CNV, MSI, and Ploidy between two cancer subtypes.

We calculated the Jaccard similarity coefficient (Jaccard Index) (Bu et al., 2021b) of two tumors in the sample patient based on the analysis of shared mutations. An index of 0.01 was taken as the screening threshold, and 17 patients were divided into two groups in total. Among them, we defined those with index <0.01 as separate primary hepatocellular carcinoma, 10 cases in total, and those with index >0.01 as metastatic, 7 cases in total (Figure 2A). The index of the metastatic group ranged from 0.08 to 0.7. The analysis of PFS showed that patients with metastatic pattern showed worse survival (*p*-*value* = 0.1142) (Figure 2B). Analysis of differences in mutations between the two subgroups showed that TP53 was more inclined to be present in the subgroup with the metastatic pattern, with a *p-value* of 0.0212 (8/14 vs. 1/20). ARID1A had a slight elevation in metastasis, with *p*-*value* = 0.2022 (4/14 vs. 2/20) (Figure 2C). The analysis of the difference among TMB, CNV, MSI, and Ploidy showed a slight increase in TMB (*p-value* = 0.1199, average = 6.35 vs. 5.44) and a significant increase in CNV (*p-value* = 0.0327, average = 358.13 vs. 225.68) in the metastatic group. At the same time, there was no significant difference between MSI and Ploidy (Figure 2D).

### Phylogenetic Analysis of Hepatocarcinogenesis

According to Jaccard’s similarity coefficient, seventeen individuals were divided into two groups, of which seven were branching evolutionary (metastatic) patterns, and ten were independent occurrence patterns (Supplementary Figure S1). We further used phylogenetic tree analysis to show the evolutionary patterns of different cancer lesions and discover essential driver genes. The results shown by the phylogenetic tree were consistent with the Jaccard similarity coefficient.

**FIGURE 3:**
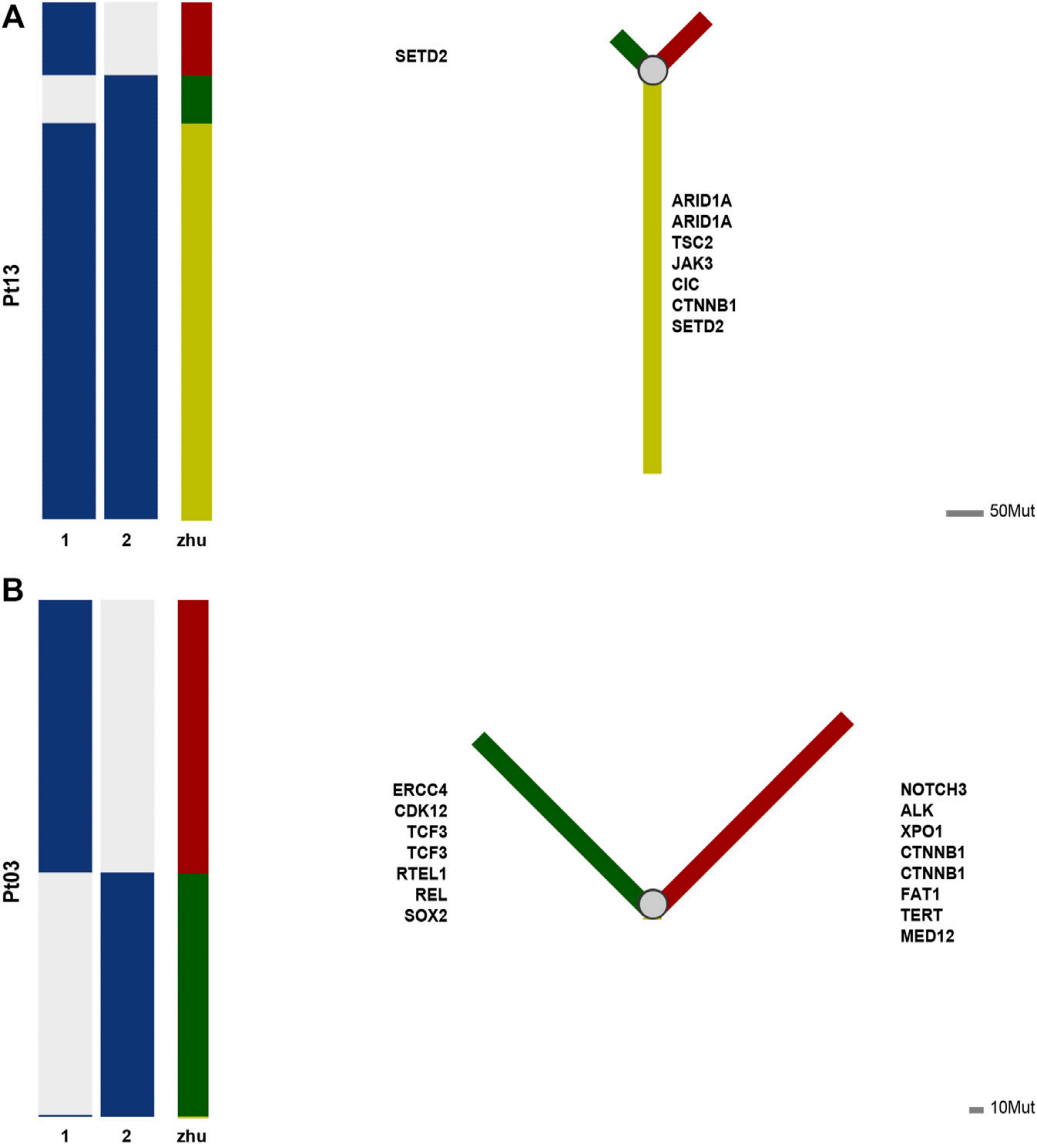
**(A)** Phylogenetic tree of patient Pt13. **(B)** Phylogenetic tree of patient Pt03.

For example, in the case of Pt13, the two tumors are highly similar according to the Jaccard similarity coefficient. Moreover, according to the results of the phylogenetic tree, a total of 107 mutations occurred in the two lesions, of which 82 mutations were shared in both samples (76.6%), i.e., located in the branching part of the shared phylogenetic tree (Figure 3A). Among them, ARID1A, TSC2, JAK3, CIC, CINNB1, and SETD2 were mutated at the early stage of carcinogenesis, which played an essential role in advancing early cancer development and progression.

In contrast, case of Pt03 had low level of similarity between the two tumors. As shown by the phylogenetic tree, 345 mutations occurred in either of tumor, while only TNIP2 was a shared early mutation (Figure 3B). And TNIP2 is less reported in cancer and is more like passenger mutation, so the mutation sharing here may be due to technical bias of sequencing or some accumulated alterations due to HBV infection. The phylogenetic trees of remaining cases are available at Supplementary Figures S1–S5.

### Pathological Cross-Analysis

The results of molecular testing can provide precise results for accurate diagnostic typing. However, more accessible in the clinic, pathology testing require simpler processing and short time consuming than molecular testing. Therefore, we attempted to compare pathology results from different individuals to determine what percentage of molecular typing could be consistently distinguished by pathology typing. In simple words, assuming molecular typing results as the standard, we wanted to see how much of a typing indication the pathology could achieve. From there, we can determine the scenario in which pathology and molecules are used in combination with each other.

**FIGURE 4:**
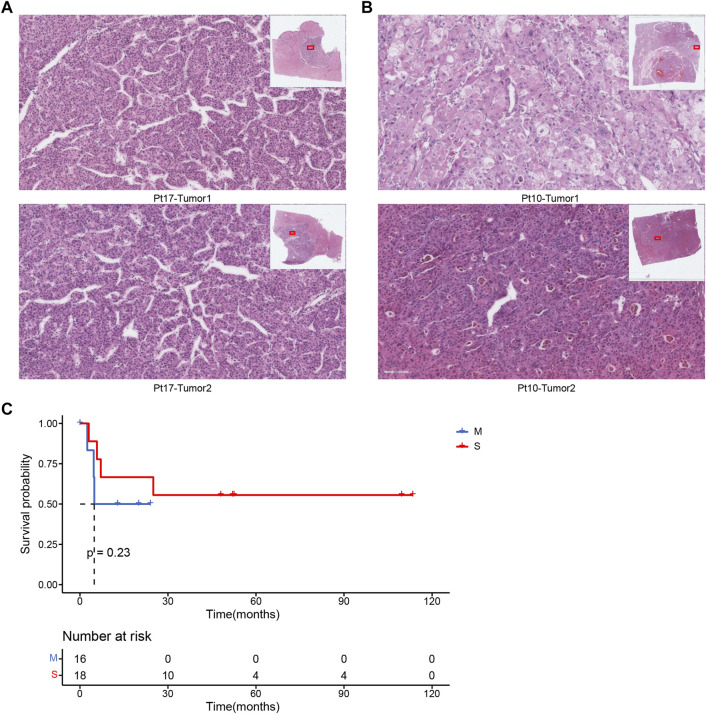
**(A)** Pathological section of patient Pt17. **(B)** Pathological section of patient Pt10. **(C)** Associations between cancer types by pathologists and OS.

We had a mid-level pathologist interpret the pathological images of these 17 patients and then compared the results of pathological typing with those of molecular testing. The analysis of the results of the 17 cases showed that 70.6% (12 cases) of the molecular typing results could be distinguished by pathological indicators, using the typical indicators of the nuclei of the pathological sections as important measures. Case Pt17 was classified as metastatic by pathological typing because the cell morphology of the two tumors was very similar (Figure 4A). This result is consistent with the results of molecular typing. In contrast, case Pt10 was classified as the seperate primary HCC because the cell morphology of the two tumors was quite different, such as the cellular atypia and sinusoids (Figure 4B). In addition, the survival analysis results of pathological typing showed that pathological interpretation could slightly distinguish between the two cancer subtypes (Figure 4C).

**FIGURE 5:**
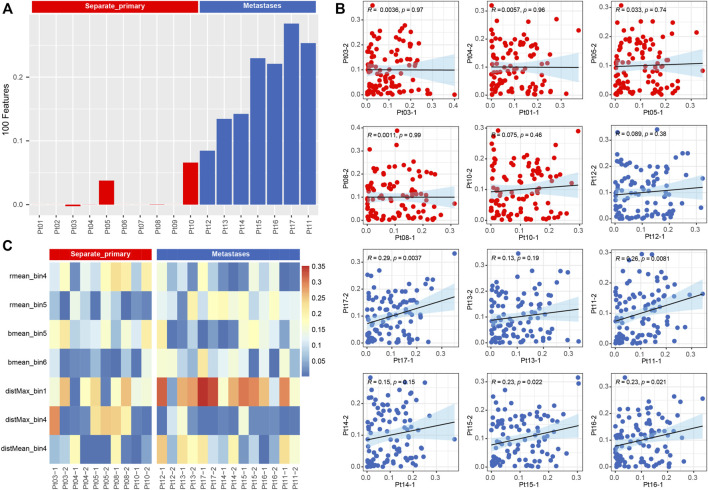
**(A)** Barplot of correlation from all pathological image feature. **(B)** Boxplot of correlation from 15 types of pathological image feature.

Meanwhile, we used a machine learning approach (Cheng et al., 2020) to extract 100 features of the pathological images, represented by matrix vectors, to discriminate between two subtypes by comparing the pathological features of two foci slides. First, all features were combined to calculate the correlation between the two foci of the same patient (Figure 5C), and the results showed that the subtypes could be distinguished by correlation (*p* < 0.001) (Figure 5A). Second, all 100 pathological features were compared between two groups, and we found that the features of rmean_bin4, rmean_bin5, bmean_bin5, bmean_bin6, disMax_bin1,disMax_bin4 and distMean_bin4 are significantly different between the two groups (Figure 5B).

## Discussion

Hepatocellular carcinoma is a cancer with a high degree of malignancy (Chidambaranathan-Reghupaty et al., 2021). In this study, we collected a cohort of 17 patients with multifocal hepatocellular carcinoma. Then we utilized the bioinformatics approach to analyze the whole-exome molecular data and image data of H&E stained histology slides. By calculating the Jaccard Index between two tumor and reconstructing the tumor clonal evolution, we revealed that intrahepatic metastasis and separate primary patterns reflected from the unique gene mutations and copy number alterations. We also utilized a machine learning approach to extract 100 features of the pathological images, to discriminate between two subtypes by comparing the pathological features of two focal H&E slides.

As two standard approaches for accurate diagnosis in the clinic, we explored the consistency between molecular testing and pathological testing. We confirmed that the pathology results could have 70.5% agreement with those of molecular testing. Based on these results, we propose a multi-modality way to differentiate the pattern of multifocal hepatocellular carcinoma using molecular or pathology testing in different clinical scenarios to provide guidelines for diagnosing and treating multifocal hepatocellular carcinoma.

Due to the scarcity of samples for multifocal hepatocellular carcinoma, only 34 samples were collected in this study, which may limit our construction of a more effective mathematical model for molecular subtyping. We may not achieve a significant outcome if the sample size is not large enough. Therefore, in this study, we mainly took a differential comparison to discover possible molecular biomarkers, and analyzed molecular and clinical features to explore how well the molecules testing is consistent with the pathology testing. Following this work, we are conducting a clinical study of multifocal HCC, yielding a more extensive data collection in the future. We will use advanced computational techniques such as artificial intelligence to optimize further the mathematical model of molecular typing (Tanaka et al., 2021), and some biological intelligent interpreters (Bu et al., 2021a) to generate multiple biomedical knowledge. Moreover, decision tools with multimodal combinations (Patel et al., 2021) could also be developed to optimize the diagnosis of multifocal HCC and thus guide the clinical treatment of liver cancer.

## Data Availability

The original contributions presented in the study are included in the article/Supplementary Material, further inquiries can be directed to the corresponding authors.

## Supplementary Material

Supplementary Figures S1–S5 The phylogenetic tree of the remaining patients except for patient Pt13 and Pt03.

Supplementary Table S1 The detailed clinical pathological information of all patients.

Supplementary Table S2 The detailed somatic mdutation information of all samples.
