# Supplementary figures and images for: Cross Analysis of Genomic-Pathologic Features on Multiple Primary Hepatocellular Carcinoma
Source: Front Genet. 2022 Jun 20;13:846517. doi: 10.3389/fgene.2022.846517 (PMC9251469; doi:10.3389/fgene.2022.846517)

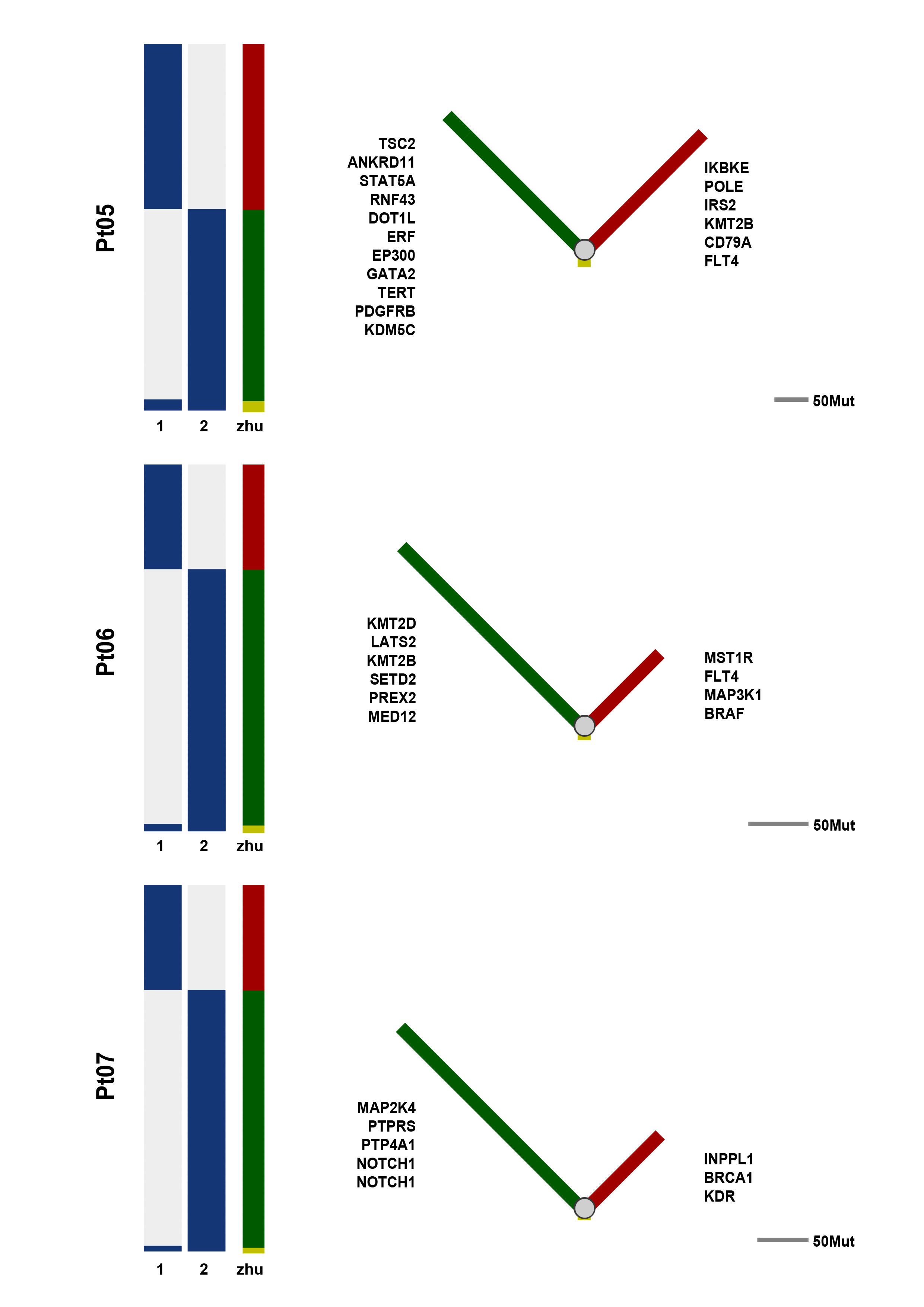

Supplement: Supplementary file 2 [file Image3.TIF]

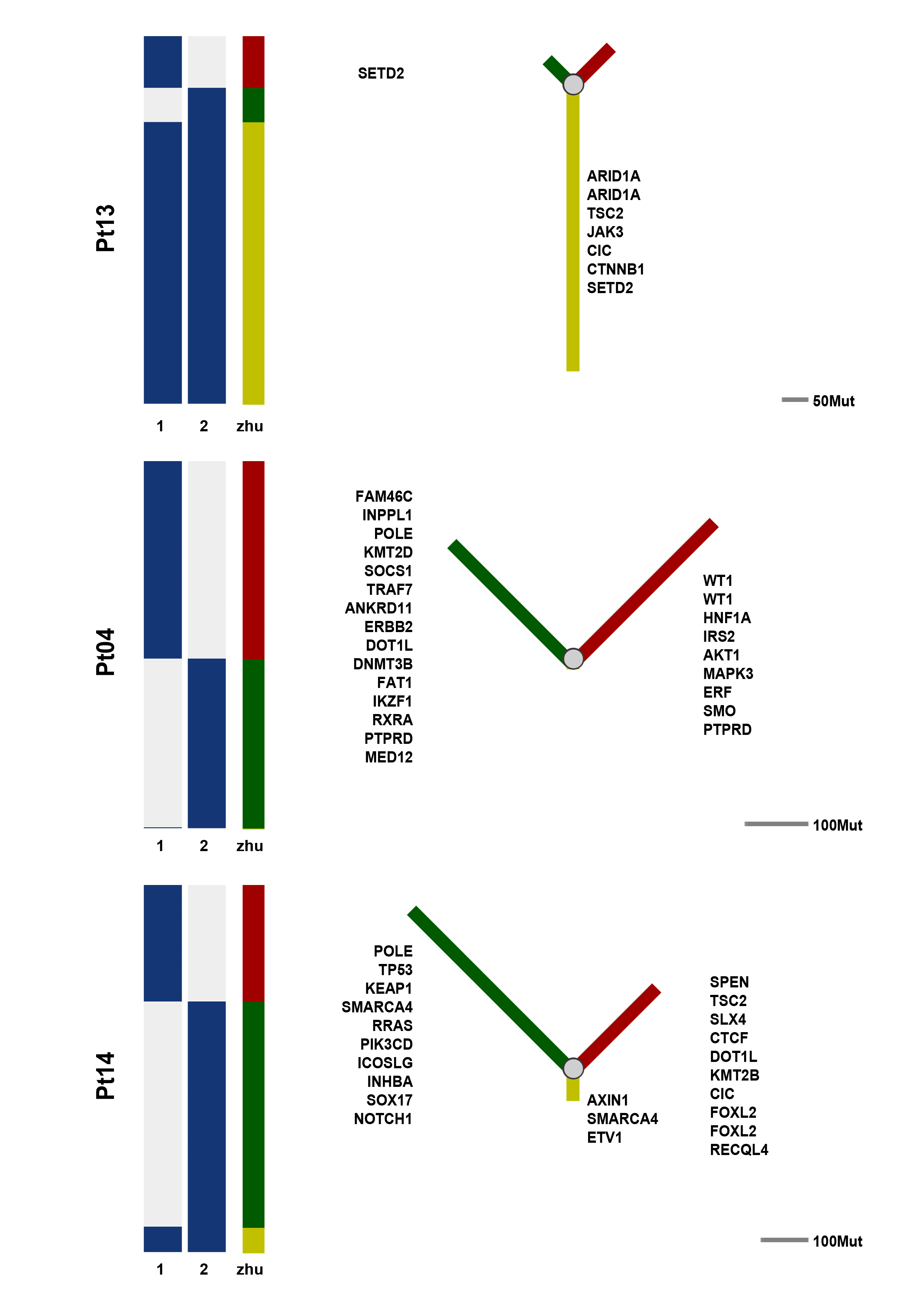

Supplement: Supplementary file 3 [file Image4.TIF]

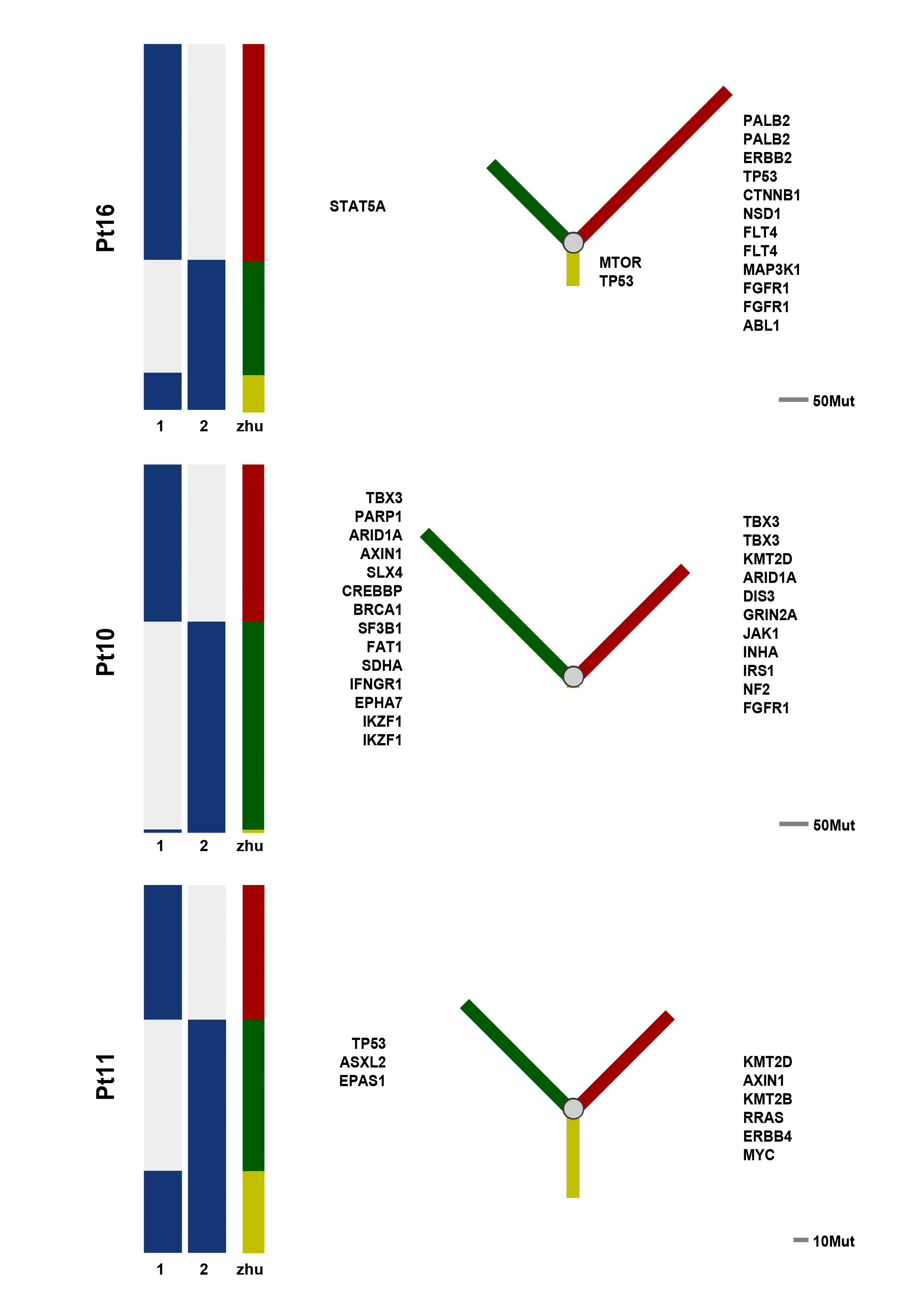

Supplement: Supplementary file 4 [file Image2.TIF]

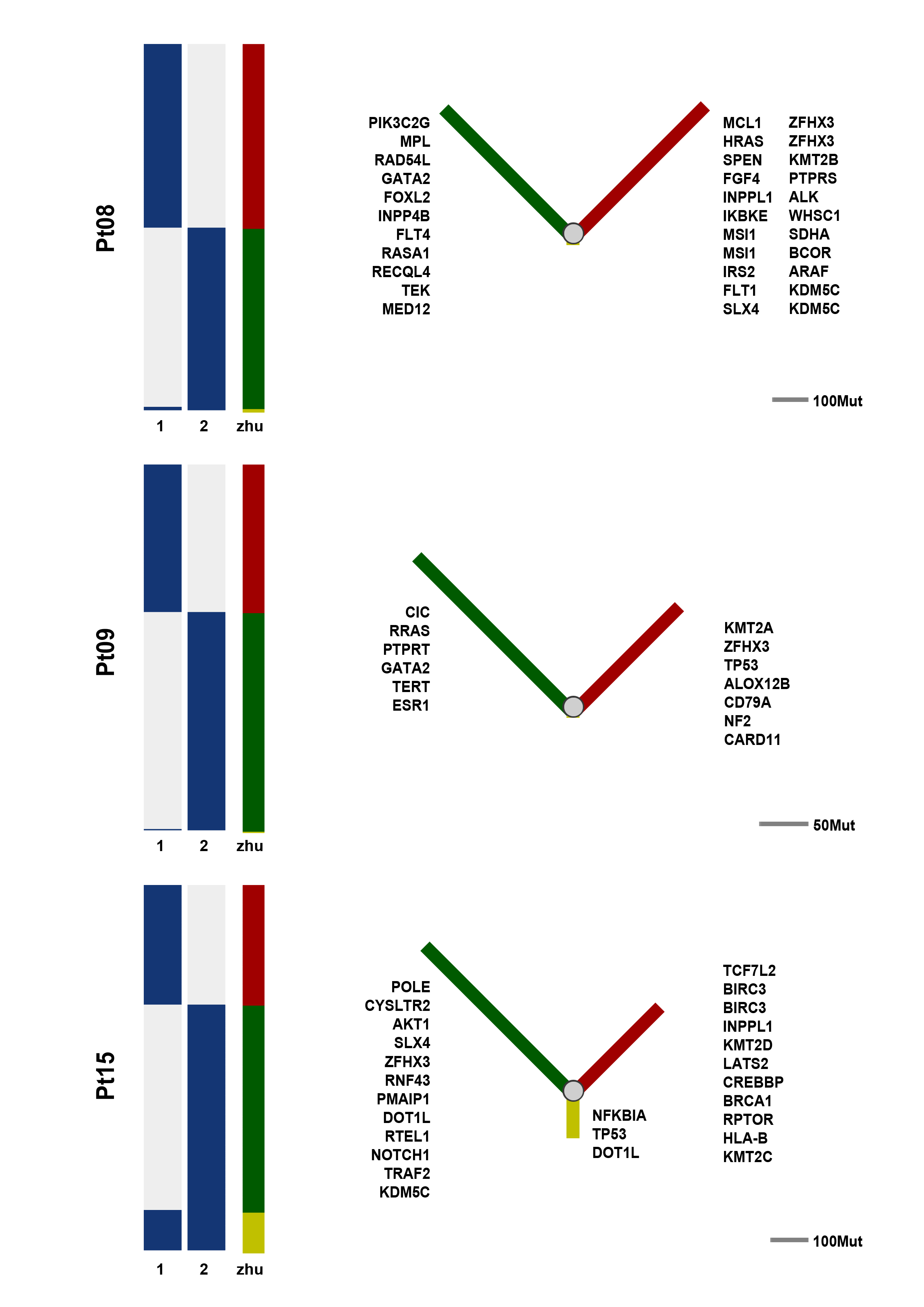

Supplement: Supplementary file 5 [file Image1.TIF]

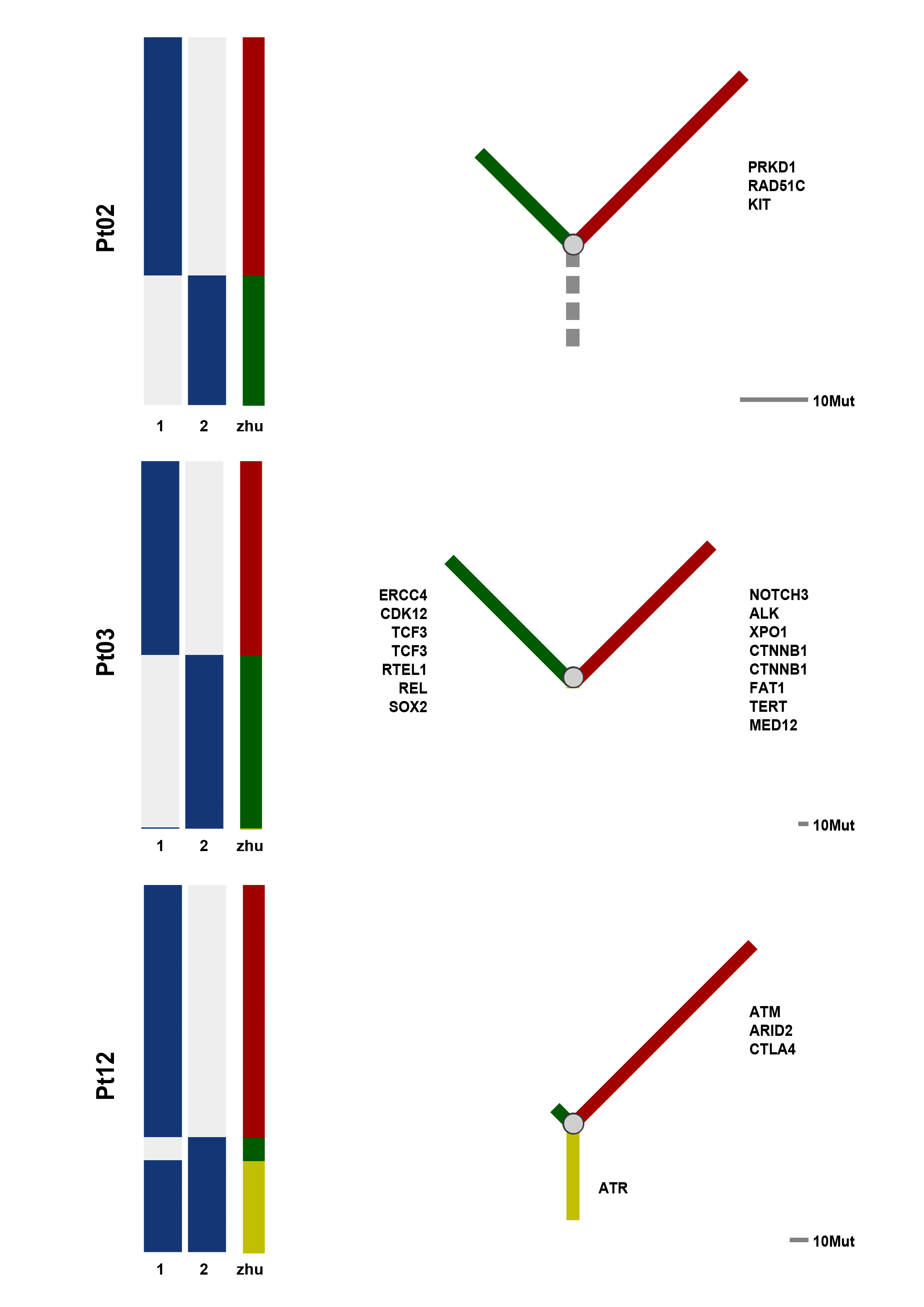

Supplement: Supplementary file 7 [file Image5.TIF]
